# Reliability and Validity of the Geriatric Depression Scale in a Sample of Portuguese Older Adults with Mild-to-Moderate Cognitive Impairment

**DOI:** 10.3390/brainsci13081160

**Published:** 2023-08-03

**Authors:** Susana I. Justo-Henriques, Enrique Pérez-Sáez, Janessa O. Carvalho, Elzbieta Bobrowicz-Campos, João L. Alves Apóstolo, Patricia Otero, Fernando L. Vázquez

**Affiliations:** 1 Health Sciences Research Unit, Nursing (UICISA:E), Nursing School of Coimbra (ESEnfC), 3004-011 Coimbra, Portugal; 2 National Reference Centre for Alzheimer’s and Dementia Care, Imserso, 37008 Salamanca, Spain; 3 Department of Psychology, Bridgewater State University, Bridgewater, MA 02325, USA; 4 Centre for Psychological Research and Social Intervention, Iscte-University Institute of Lisbon, 1649-026 Lisboa, Portugal; 5 Department of Psychology, University of A Coruña, 15071 A Coruña, Spain; 6 Department of Clinical Psychology and Psychobiology, University of Santiago de Compostela, 15782 Santiago de Compostela, Spain

**Keywords:** depressive symptoms, older adults, geriatric depression scale, depression screening

## Abstract

Although the Geriatric Depression Scale (GDS) is a well-established instrument for the assessment of depressive symptoms in older adults, this has not been validated specifically for Portuguese older adults with cognitive impairment. The objective of this study was to analyze the psychometric properties of two Portuguese versions of the GDS (GDS-27 and GDS-15) in a sample of Portuguese older adults with mild-to-moderate cognitive impairment. Clinicians assessed for major depressive disorder and cognitive functioning in 117 participants with mild-to-moderate cognitive decline (76.9% female, M_age_ = 83.66 years). The internal consistency of GDS-27 and GDS-15 were 0.874 and 0.812, respectively. There was a significant correlation between GDS-27 and GDS-15 with the Beck Depression Inventory-II (GDS-27: rho = 0.738, *p* < 0.001; GDS-15: rho = 0.760, *p* < 0.001), suggesting good validity. A cutoff point of 15/16 in GDS-27 and 8/9 in GDS-15 resulted in the identification of persons with depression (GDS-27: sensitivity 100%, specificity 63%; GDS-15: sensitivity 90%, specificity 62%). Overall, the GDS-27 and GDS-15 are reliable and valid instruments for the assessment of depression in Portuguese-speaking older adults with cognitive impairment.

## 1. Introduction

Population ageing is occurring globally at an unprecedented rate and will accelerate in the coming decades, particularly in developing countries [1]. The World Health Organization estimates that the number of older adults aged 60 and over will reach up to 2.1 billion worldwide by 2050, with nearly 80% stemming from low- and middle-income countries [2]. According to data from the Statistics National Institute, older adults in Portugal comprise approximately 21.5% of the population, with an expected increase to 37.2% by 2080 [3].

Older adults, without normal ageing, are particularly vulnerable to mental and neurological health conditions such as neurocognitive disorders and depression due to additional stress factors such as loss of capacity [4]. The effects of depression can be chronic or recurrent and can dramatically affect an individual’s daily functioning and quality of life [1]. Additionally, when depression is associated with chronic illness, it increases morbidity and mortality, leading to psychological and financial burden on the individual, family, and health system [5]. Thus, the health status of older adults has significant personal, community, and national impacts. Depression often accompanies neurocognitive disorders, is a risk factor and prodrome, and may indicate worse prognosis [6]. Therefore, early and timely identification of mood changes may be crucial in treating cognitive functioning decline. Thus, it is crucial to have screening tools for depression that are suitable for use in older adults with cognitive impairment.

The clinical manifestations of depression in older adults are complex as they involve biological, psychological, and social aspects, often related to lifestyle changes and cognitive decline [7]. Identifying depression in the context of primary care, particularly in patients with multiple comorbidities, also can be difficult. As a consequence, depression is often under-diagnosed and under-treated in older adults [8]. Therefore, self-response questionnaires have emerged as an approach to help primary care providers identify patients who may have depression but do not yet have a diagnosis [4].

Two existing gold-standard instruments for assessing depression throughout the lifespan include the Beck Depression Inventory-II (BDI-II), originally developed by Beck et al. [9], and for older adults, the Geriatric Depression Scale (GDS) created by Yesavage et al. [10,11]. The GDS is a widely used tool for depression screening, specifically designed for the elderly. This instrument does not contain somatic symptomatology assessment items (unlike other depression screening tools) on the grounds that these may lack discriminatory capacity in older adults because they can be attributed to comorbid physical conditions and the ageing process [10]. Additionally, the format of the GDS includes dichotomous yes/no items rather than the multi-level items in the BDI-II and has a reduced retrospective recall time span (1 week for the GDS versus 2 weeks for the BDI-II) making it a more simplified tool for older adults. Therefore, the GDS can be administered to older adults regardless of physical illness or cognitive impairment [12] and is a reliable screening tool for depressive symptoms in mild cognitive impairment [13] and dementia [13,14,15]. However, as shown by a recent systematic review [16], research focused on the accuracy of this measure for screening of depression in older adults with cognitive impairment is still sparse and further studies are needed to enable the selection of optimal cutoff values. 

The original version of GDS has thirty items (GDS-30), though shorter forms (fifteen items (GDS-15); ten items (GDS-10); four items (GDS-4)) have been developed [10]. These versions have been translated in multiple languages (e.g., Durmaz et al. [17]; Galeoto et al. [18]; Sugishita et al. [19]), including Portuguese [20,21,22,23,24], which is the primary focus of the current study. Systematic reviews of the Portuguese-translated briefer versions have been conducted to determine their diagnostic accuracy, validity, and reliability with good support for both in these versions [12,25,26,27,28]. The conclusion was that shorter versions of the GDS can be administered for depression screening in primary care and in a community setting and are more efficient than longer forms that carry redundant items [12,25,26,29]. 

In Portugal, the GDS-30 was adapted and validated by Pocinho et al. [20], while a 15-item (GDS-15) version was adapted and validated by Apóstolo et al. [21,22]. Both versions demonstrated good psychometric properties; hence, they present with potential as sound instruments for screening for depressive symptoms in older adults. However, these have not been validated specifically in samples of Portuguese individuals with cognitive impairment, which is important to explore as comprehension of items and response styles may differ in those with cognitive difficulties, thus affecting the outcomes/interpretation. 

The aim of this study was to explore the psychometric properties (namely, internal consistency, reliability, and construct validity) of two Portuguese versions of the GDS (GDS-30 and GDS-15) in a Portuguese mild-to-moderate cognitive impairment sample and to compare their performance to the DSM-5’s diagnostic criteria for major depressive episode (thus exploring criterion validity). Establishing reliability and validity, two critical psychometric properties, is critically important for accurate and interpretable assessment [30]. Thus, this is a critical mechanism that needs to be explored in various populations and instruments to instill confidence in the measured outcome and drives the importance of the current study. The influence of sociodemographic variables—namely, age, education level, and gender—on the performance of two GDS versions were also analyzed. Notably, we used the GDS-27 version in the current study as modified in Pocinho et al. [20], in which item-level performance revealed that three items on the original GDS-30 (items 27, 29, and 30) were found to be weak in the Portuguese translation; thus, our version reflected the psychometrically stronger (and more commonly administered) GDS-27 version. 

## 2. Methods

### 2.1. Participants

This cross-sectional study was conducted on a convenience sample of 161 older adults recruited through 12 institutions that provide social care and support services for older adults (including people living in long-term care centres, people attending day and social centres, and people receiving home support services), located in urban (one in the northern region and four in the central region) and rural (five in the central region and two in the southern region) areas of Portugal. Inclusion criteria included the following: (a) aged 65 years or over; (b) diagnosed with neurocognitive disorder by a clinical psychologist as per DSM-5 criteria [31]; (c) able to engage and understand the assessment questions; and (d) native Portuguese speaker. 

Exclusion criteria included older adults that had severe sensory and/or physical limitations, were not oriented to the environment, or had severe neuropsychiatric symptoms that prevented the completion of the assessment instruments. Another exclusion criterion took into account Mini Mental State Examination (MMSE; see details in Instruments section) scores. As our primary focus was on mild-to-moderate cognitive impairment, participants with severe cognitive impairment (MMSE score of less than 9) were not included, nor were those without evidence of cognitive impairment (MMSE score of 22 or better for those with 0 to 2 years of formal education; 24 or better for those with 3 to 6 years of formal education; 27 or better for those with 7 or more years of formal education). Of the 161 persons contacted to participate in the study, 44 were excluded based on MMSE scores, resulting in a final sample of 117 participants. The study was conducted in accordance with the latest version of the Declaration of Helsinki and was approved by the Ethics Committee of the Health Sciences Research Unit: Nursing, part of the Nursing School of Coimbra (code number P629/11-2019). Prior to the inclusion of the subjects in the study, signed informed consent was obtained from the participants or their legal representatives. This information included the processing of data in accordance with current legislation, the voluntary nature of participation in the study, the participants receiving no financial compensation or any other incentive, and the right to withdraw consent at any time, without affecting the services received at the institution. Additionally, throughout the study, the evaluators monitored the participants for indications that they did not wish to participate in the evaluations.

### 2.2. Instruments

A self-reported sociodemographic and clinical questionnaire was administered, collecting data on gender, age, marital status, educational level, type of institution attended, presence of medical comorbidities, and cognitive symptoms. In addition, the following self-report assessment tools were used:

Geriatric Depression Scale-30 (GDS-30): full version, with reported good reliability (Cronbach’s alpha = 0.91). Items are presented in dichotomous format (yes/no). Score range 0–30. Scores of 11 or more out of a maximum of 30 points are suggestive of clinical depression. In the present study, the 27-item version was used. It is easy to administer and is indicated for administration to people suffering from cognitive decline [10,20]. 

Geriatric Depression Scale-15 (GDS-15): A 15-item version of the GDS with good reliability (Cronbach’s alpha = 0.83). It consists of 15 questions in a dichotomous format (yes/no). Score range 0–15. Scores equal to or greater than 6 out of a maximum of 15 points are considered to be indicators of depression [21,32]. The authors suggest that this tool is effective for screening for depression in older adults [22].

Beck Depression Inventory-II (BDI-II): One of the most widely used tools for assessing self-reported depression, presenting with good reliability (Cronbach’s alpha = 0.90) [33,34]. It consists of 21 items that assess symptoms characteristic of depression during the last two weeks. It consists of a cognitive–affective scale (items 1–10, 12, 14, and 21) and a somatic scale (items 11, 13, 16–20). Each item is scored from 0 to 3, with 0 reflecting the absence of the symptom and a higher value reflecting greater symptom severity. The overall score ranges from 0 to 63 points. There is evidence that the BDI-II is a reliable and valid tool to be used in screening for depression in older adults, including those with cognitive impairment [35], and that it has practical utility in different healthcare contexts [36,37,38].

The MMSE (Folstein et al. [39]; Portuguese version by Guerreiro et al. [40]) is widely known and used as a screening tool of cognitive function evaluation, interpreted based on normative data for different literacy groups established by Morgado et al. [41]. Score range 0–30. The cutoff points used followed the normative data established by Morgado et al. [41] and took into account three literacy groups. Namely, in those with 0 to 2 years of formal education, the cutoff point of 22 was used; in those with 3 to 6 years of formal education, the cutoff point of 24 was used; in those with 7 or more years of formal education, the cutoff point of 27 was used. The Portuguese version by Guerreiro et al. [40] shows good reliability (Cronbach’s alpha = 0.89).

We also administered a module of the Structured Clinical Interview for the Disorders of the DSM-5, Clinician Version (SCID-5-CV) [42], which is based on the DSM-5 criteria [31] for depressive disorders, with inter-observer reliability (κ) ranging from 0.70 to 1.00.

### 2.3. Procedures

The sample was recruited via non-probabilistic sampling among 12 institutions that provide social care and support services for older adults (including people living in long-term care centres, people attending day and social centres, and people receiving home support services), located in urban and rural areas of Portugal. Older adults with cognitive impairment and their legal representatives were contacted, the details of the study were explained to them, and they were invited to participate.

A psychologist with two or more years of experience and familiarity with the measures used in the study asked participants who met the inclusion criteria to complete a sociodemographic questionnaire, the Portuguese version of the GDS-27 scale, the Portuguese version of the GDS-15 scale, and the BDI-II (which was used as the gold standard to assess symptoms of depression). In the case where the participants had less reading experience, reading the questions and recording the answers obtained was the responsibility of the professional. A clinical psychologist with two or more years of experience, who had previously undergone training of at least 4 h, administered the module for major depression of the SCID-5-CV. The clinician was knowledgeable about the results of the GDS and BDI. 

The instruments were given in a single session and the order of administration was as follows: (1) MMSE, to determine whether they met inclusion criteria of diagnosis of neurocognitive disorder; (2) depression screening instruments; and (3) the SCID-5-CV.

### 2.4. Statistical Analysis

To compare the distribution of categorical variables in independent groups, the Chi-Squared test was used, with effect size being estimated based on the Phi (φ) statistic for 2 × 2 contingency tables or on Cramer’s *V* statistic for non 2 × 2 contingency tables. To compare the variance of ordinal variables in independent groups, the Mann–Whitney test (for two groups) and the Kruskal–Wallis test (for more than two groups) were used due to non-normal distribution of the results obtained. In relation to the Mann–Whitney test, the effect size was calculated based on a formula “r = Z/√N” and interpreted based on the indications proposed by Cohen [43]. Regarding the Kruskal–Wallis test, if differences were statistically significant, a multiple comparison of mean ranks was performed. The effect size was calculated using the Partial Eta Squared measure (η^2^_p_) and interpreted following Cohen’s [43] and Marôco’s [44] suggestions. 

Internal consistency of GDS questionnaires was measured using the Kuder–Richardson coefficient, KR-20, which is indicated for dichotomous variables [45]. Additionally, McDonald’s omega Ὠ was calculated as this coefficient proved to be a robust alternative for cases in which the assumptions for the use of Cronbach’s alpha (such as unidimensionality and absence of normality violations) are not met [46]; although, the use of McDonald’s omega is mainly recommended for large samples. Congruent validity was determined through the Spearman correlation coefficients, calculated for GDS questionnaires and BDI-II. 

To analyze the possible effect of covariates on dependent variables, a non-parametric ANCOVA was performed using the *F* statistic calculated according to the Quade Method. The Quade Method involves testing the equality of the residuals between groups obtained through linear regression of the ranked dependent variable on the ranked covariate. 

The two-factor interaction effect on the dependent variables was also analyzed. For this purpose, a non-parametric two-way ANOVA was performed, using the *H* statistic calculated based on the formula in which the sum of the squared ranks of a given factor is divided by the total mean square for those ranks. The effect size was indicated by the η^2^_p_ coefficient.

The receiver operating characteristic (ROC) curve for GDS scores and DSM-5 diagnosis (present/absent) was also plotted to establish the sensitivity and specificity of different cut-off points for depression screening. The selection of an optimal cut-off point considered the maximum Youden index, calculated according to the formula “sensitivity + specificity − 1” [47]. Other standard summary measures of test accuracy, such as positive and negative predictive values, were also calculated.

In all analyses, a statistical significance level of 0.05 was considered. For data treatment, the Statistical Package for Social Sciences (IBM SPSS Statistics, New York, NY, USA), version 25.0, was used.

## 3. Results

### 3.1. Sample Profile

From the 117 older adults presented in Table 1, applying the DSM-5 diagnostic criteria for a major depressive episode, 20 participants were classified as having depression and 97 as not having depression; among the depressed participants, 10 met the criteria for a mild depressive episode, seven for a moderate depressive episode, and three for a severe depressive episode. There were no significant differences between depressed and non-depressed participants in terms of mean age (U(97, 20) = 958.50, *p* = 0.934), male/female ratio (χ^2^(1) = 0.887, *p* = 0.346), and formal education level ratio (χ^2^(2) = 5.106, *p* = 0.078). The groups were also equivalent in terms of MMSE score (U(97, 20) = 961.00, *p* = 0.948).

### 3.2. Depressive Symptomatology and Sociodemographic Characteristics

The mean GDS-27 and GDS-15 scores obtained in this sample were 14.11 (SD = 6.68) and 7.68 (SD = 3.75), respectively. Analyses of the potential effect of age, gender, and formal education level on the distribution of sociodemographic, clinical, and neuropsychological characteristics of the participants in the GDS-27 and GDS-15 scores in depressed and non-depressed participants were also performed. To analyze the influence of age, a non-parametric ANCOVA was performed using mean ranks of both the dependent variable and covariate. The F statistic was calculated using the univariate ANOVA of non-standardized residuals obtained through linear regression of the rank of the dependent variable on the rank of the covariate. The F-test showed that the different scores obtained by depressed and non-depressed older adults in both questionaries cannot be explained by age distribution (GDS-27: F = 29.657, *p* < 0.001, η^2^_p_ = 0.205; GDS-15: F = 24.817, *p* < 0.001, η^2^_p_ = 0.177).

The potential influence of gender on the variables of interest was analyzed based on a two-factor non-parametric ANOVA. The interaction between the gender group (male/female) and depression group (present/absent) was not statistically significant, independently of the questionnaire used (GDS-27: H(1) = 0.666, *p* = 0.414; GDS-15: H(1) = 0.147, *p* = 0.701). In terms of main effects, the depression group-related factor was shown to contribute to the GDS scores distribution (GDS-27: H(1) = 9.215, *p* = 0.002, η^2^_p_ = 0.091; GDS-15: H(1) = 9.584, *p* = 0.002, η^2^_p_ = 0.092); the same was not verified for the gender group-related factor (GDS-27: H(1) = 0.259, *p* = 0.610; GDS-15: H(1) = 0.001, *p* = 0.969). The two-factor non-parametric ANOVA was also used to analyze the potential effect of formal education level on the GDS score distribution in the two participant groups. In both GDS questionnaires, the interaction between the literacy group (0–2 years of formal education/3–6 years of formal education/7 or more years of formal education) and depression group (present/absent) showed no influence on the score distribution (GDS-27: H(2) = 0.040, *p* = 0.841; GDS-15: H(2) = 0.019, *p* = 0.891). In terms of main effects, the distribution of the GDS score was proved to be influenced by the depression group-related factor (GDS-27: H(1) = 21.592, *p* < 0.001, η^2^_p_ = 0.195; GDS-15: H(1) = 19.042, *p* < 0.001, η^2^_p_ = 0.174) but not by the literacy group-related factor (GDS-27: H(2) = 2.672, *p* = 0.263; GDS-15: H(2) = 4.182, *p* = 0.124). 

### 3.3. Reliability

The reliability of GDS-27 and GDS-15 was calculated based on the responses of 94 participants (18 with depression and 76 without depression). With regard to the GDS-27, the item means ranged from 0.27 (item 15) to 0.80 (item 17). The item-total correlations were found to be strong (r ≥ 0.70) for items 4 and 16, and weak (*r* ≤ 0.40) for items 12, 14, 15, 18, 22, and 28. In the case of the remaining 19 items, the item-total correlations were revealed to be moderate (0.40 < r < 0.70). The corrected item-total correlations ranged between 0.13 (item 12) and 0.74 (item 4).

Regarding the GDS-15, the item means ranged from 0.21 (item 11) to 0.77 (item 3). The item-total correlations were shown to be strong (r ≥ 0.70) for items 1 and 4, and weak (r ≤ 0.40) for items 6, 9, 10, and 11. The remaining nine items correlated moderately (0.40 < r < 0.70) with the total. The corrected item-total correlations ranged between 0.14 (item 9) and 0.65 (item 4).

Internal consistency estimated by KR-20 and Ὠ was shown to be high for both versions (GDS-27: KR-20 = 0.874, Ὠ = 0.868; GDS-15: KR-20 = 0.812, Ὠ = 0.810). Internal consistency did not change substantially with the deletion of items (GDS-27: KR-20 if item deleted ranged between 0.861 and 0.877, Ὠ if item deleted ranged between 0.852 and 0.871; GDS-15: KR-20 if item deleted ranged between 0.784 and 0.822, Ὠ if item deleted ranged between 0.780 and 0.820). 

### 3.4. Relationship between GDS and Other Measures

#### 3.4.1. Convergent Validity

The GDS-27 and GDS-15 scores correlated strongly with the BDI-II total score (GDS-27 × BDI-II: rho = 0.738, *p* < 0.001; GDS-15 × BDI-II: rho = 0.760, *p* < 0.001). Significant correlations were also found between the scores obtained on the GDS questionnaires and on the BDI-II cognitive–affective and somatic scales; these were of strong and moderate magnitude (GDS-27 × BDI-II cognitive–affective scale: rho = 0.711, *p* < 0.001; GDS-27 × BDI-II somatic scale: rho = 0.613, *p* < 0.001; GDS-15 × BDI-II cognitive–affective scale: rho = 0.747, p < 0.001; GDS-15 × BDI-II somatic scale: rho = 0.669, *p* < 0.001). 

#### 3.4.2. GDS-27 and GDS-15 Scores in Depressed and Non-Depressed Participants

On both GDS questionnaires, non-depressed participants scored lower than depressed ones. The differences observed were statistically significant (GDS-27: U(97, 20) = 298.00, *p* < 0.001, r = 0.45; GDS-15: U(97, 20) = 347.00, *p* < 0.001, r = 0.42). The analyses considering non-depressed participants and participants with varying symptom severity also revealed significant between-group differences in the distribution of GDS scores (GDS-27: H(3) = 24.368, *p* < 0.001; GDS-15: H(3) = 21.850, *p* < 0.001). According to the pairwise multiple comparisons of mean ranks, on both questionnaires, non-depressed participants scored significantly lower than participants with mild (GDS-27: *p* < 0.001; GDS-15: *p* < 0.001), moderate (GDS-27: *p* < 0.001; GDS-15: *p* = 0.036), and severe (GDS-27: *p* = 0.004; GDS-15: *p* < 0.001) depression. Furthermore, significant differences were found between GDS-15 scores obtained by participants with mild and severe depression (*p* = 0.001) but not between participants with mild and moderate depression or between participants with moderate and severe depression. In relation to the GDS-27, the scores obtained by participants with varying depression severity did not differ significantly. The effect size calculated for both questionnaires was medium (GDS-27 η^2^_p_ = 0.210; GDS-15 η^2^_p_ = 0.188). The corresponding descriptive statistics are presented in Table 2.

#### 3.4.3. Sensitivity and Specificity of the GDS-27 and GDS-15

**Figure 1:**
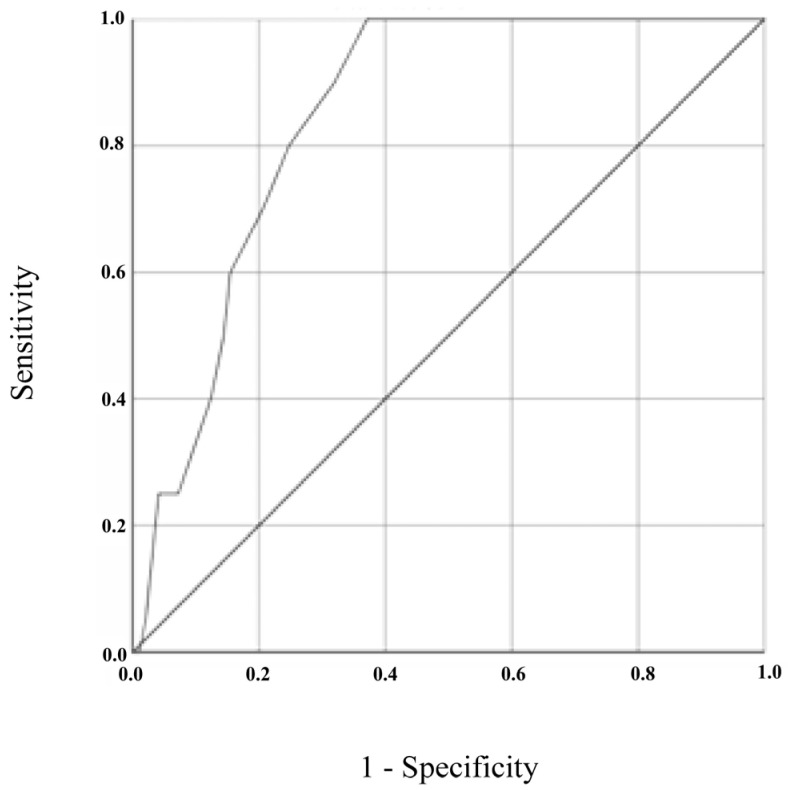
ROC curve for GDS-27, using DSM-5 diagnostic criteria as the gold standard.

**Figure 2:**
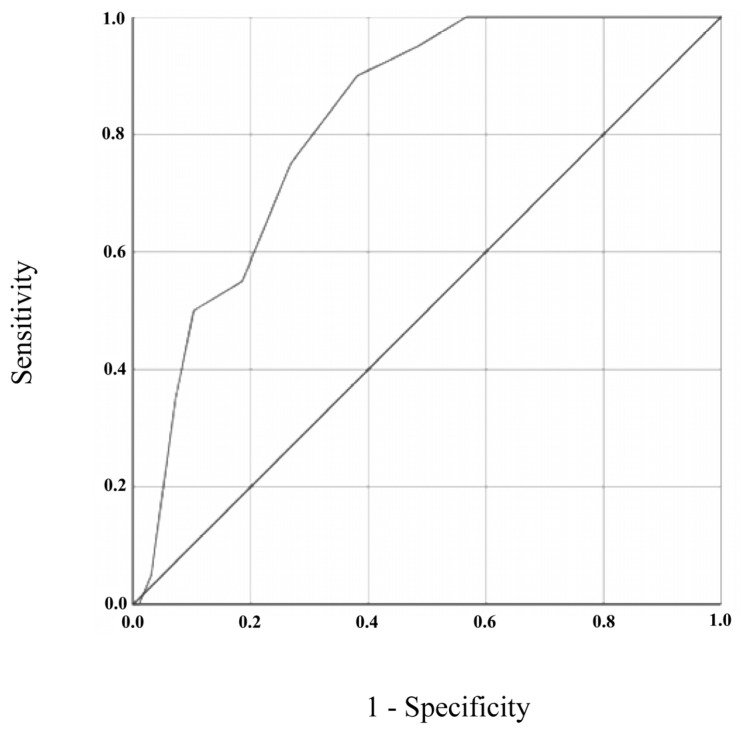
ROC curve for GDS-15, using DSM-5 diagnostic criteria as the gold standard.

Figure 1 and Figure 2 show the ROC curves for GDS-27 and GDS-15, respectively, both analyzed using the DSM-5’s diagnosis (present vs. absent) as the gold standard. The ROC curve plotted for GDS-27 revealed an AUC of 0.846 (95% CI = 0.776–0.917, *p* < 0.001). The analysis of sensitivity and specificity values and the corresponding Youden Index values, displayed in Table 3, showed an optimal cut-off for depression screening of 15/16 (absent/present), resulting in a sensitivity of 100% (95% CI = 0.80–1.00) and specificity of 63% (95% CI = 0.52–0.72). Positive and negative predictive values for a cut-off of 15/16 were 0.36 (95% CI = 0.24–0.50) and 1.00 (95% CI = 0.93–1.00), respectively.

For GDS-15, ROC curve analysis resulted in an AUC of 0.821 (95% CI = 0.739–0.904, *p* < 0.001). In this case, the optimal cut-off value for depression screening was 8/9 (absent/present), resulting in a sensitivity of 90% (95% CI = 0.67–0.98) and specificity of 62% (95% CI = 0.51–0.71). Positive and negative predictive values for a cut-off of 8/9 were 0.33 (95% CI = 0.21–0.47) and 0.97 (95% CI = 0.88–0.99), respectively.

#### 3.4.4. Depressive Symptomatology and MMSE Score

To analyze the potential effect of the covariates of MMSE score on the performance of the GDS-27 and GDS-15, a non-parametric ANCOVA was performed using mean ranks of both the dependent variable and covariate. The F statistic was calculated using the univariate ANOVA of non-standardized residuals obtained through linear regression of the rank of the dependent variable on the rank of the covariate. The F-test showed that the different scores obtained by depressed and non-depressed older adults in both questionnaires cannot be explained by the MMSE score distribution (GDS-27: F = 30.258, *p* < 0.001, η^2^_p_ = 0.208; GDS-15: F = 25.278, *p* < 0.001, η^2^_p_ = 0.180).

## 4. Discussion

This study aimed to analyze the psychometric properties and screening performance of two Portuguese-translated versions of the commonly used Geriatric Depression scale (GDS-27 and GDS-15) in older adults with mild-to-moderate cognitive impairment. The mean GDS-27 and GDS-15 scores obtained in this sample were just over 14 and 7, respectively. As expected, depressive symptoms were significantly worse for those participants who met the DSM-V criteria for depression. 

Notably, reliability analyses, performed based on Kuder–Richardson coefficient and McDonald’s omega, revealed strong internal consistency reliability in both versions of this scale (GDS-27: > 0.86; GDS-15 ≥ 0.81). These results are consistent with the internal consistency found for the general Portuguese population (GDS-27: α = 0.91; GDS-15: α = 0.83) in previous studies by Pocinho et al. [20] and Apóstolo et al. [21]. While overall internal consistency was strong, there were some items on the scale that were notably weaker than others, which raises questions about the functions of those items within our sample. For example, the lowest item correlation on the GDS-15 asks about the patient worrying about bad things happening (item 6) and the second lowest correlation asks about one’s preference to staying home versus going out and doing things (item 9). Responses to these two items in our sample, who are majority residents in a supervised institutional setting (76%), may reflect life circumstances and may be sample specific—that is, our sample mostly comprises individuals living in a supervised residential setting, which inherently have reduced autonomy and independence that may be reflected in that item (and thus, reduced variability of the item) rather than the effects of depressed mood specifically. Similar findings on the GDS-27 also were observed, with the lowest correlated items measuring preferring to stay at home rather than doing things (item 12) and being preoccupied about the past (item 18). 

Furthermore, the scale had good validity. We explored convergent validity by examining associations between both the GDS-15 and GDS-27 and the gold standard measure for self-reported depression, the BDI-II, and found strong associations. These results are consistent with previous explorations of the validity of the Portuguese GDS-30 [20] and GDS-15 [21,22] in our cognitively impaired sample. Furthermore, when grouping the BDI-II items with items aligned with cognitive–affective and somatic subscales, the two GDS versions continued to demonstrate strong associations. 

Since both the GDS-27 and the GDS-15 obtained good reliability and validity, clinicians can opt for using the GDS-15 to avoid fatigue in the population of people with cognitive impairment.

Another important indicator of a strong measure is sensitivity to identifying the underlying construct. The two GDS versions had strong sensitivity at identifying those with diagnosed clinical depression, showing its benefit as a screening tool. In particular, while this relatively brief self-report form was not strongly able to identify nuances of depression, we were able to identify those with and without diagnosed depression with high accuracy on both the GDS-15 and GDS-27. Along these lines, we were able to identify the ideal cutoff scores on the GDS scales that provide ideal levels of sensitivity and specificity. Specifically, we found scores of 8/9 on the GDS-15 and 15/16 on the GDS-27 as optimal cutoff points in the screening identification of depression. Thus, we feel confident in the ability of this tool to serve as a good screening measure that can help identify when patients need a follow-up for mood evaluation and treatment. 

Notably, our ideal cutoff scores are higher than typical cutoff points in other Portuguese-speaking populations, which fall at 4/5 on the GDS-15 [22,25]. It is possible that our cognitively impaired sample endorsed more of the cognitively oriented symptoms (e.g., more memory problems than most; worry about bad things happening) than a cognitively intact older adult population, instead capturing the effects of cognitive deficits rather than being mood-related in nature. Nonetheless, these cutoff values are still well within the ranges found in other studies. Cutoffs have been reported as high as 7/8 in other translated versions of the GDS-15 [48] and 10/11 on the GDS-30 [49]. Further, on the GDS-30, it has ranged up to 16 in other clinical samples and translations [50]. Further, while our analyses revealed high sensitivity rates (90–100%), our specificity rates were lower than statistically ideal (62–63%); however, this also is within the specificity range found in past reviews of the GDS-15 and GDS-30 [51] and, thus, consistent with the general performance of the scale. Our overall scores revealed sensitivity and specificity consistent with the GDS performance in other studies; in our sample, GDS scores remained consistent across demographic characteristic, including gender and education, and importantly arguing for good consistency of the measure. Overall, we were able to demonstrate sound discriminability of the GDS in those in our sample with and without depression.

Naturally, this study has some limitations. First, our use of a convenience sample has implications for the generalizability of the study findings to a wider population. To reduce the impact of this limitation, the recruitment process was carried out in different settings and different geographical locations, involving both institutionalized and community-dwelling older adults. Second, we used a relatively small sample size for a psychometric exploration. Namely, although a large number of older adults were contacted, the study sample included only 117 participants, which made it impossible, among other reasons, to carry out factor analysis which would have allowed a better understanding of structure of the questionnaire. The reduced adherence reflects the difficulties that health professionals often face in recruiting older adults with neurocognitive disorders to research projects. It should also be noted that of all participants included in the study, only 20 met the DSM-5 criteria for major depressive episode. Another limitation of the study was the non-use of a diagnostic tool, rather than a screening one, to complement the diagnosis of a neurocognitive disorder according to the DSM-5. Finally, because we did not control or restrict access to antidepressant medications, the present study did not evaluate whether the results obtained in the GDS were affected or not by antidepressant medication taken by participants. In further studies, this potential effect of medication intake should be examined in a controlled manner. 

## 5. Conclusions

A better understanding of the psychometrics of any measure helps improve clinicians’ and researchers’ confidence in the validity and reliability of the measure when administering this scale in their practice. This study provides support for the psychometric properties of the GDS-15 and GDS-27 in a sample of persons with mild-to-moderate cognitive impairment. Our results support that both GDS-27 and GDS-15 are reliable and valid instruments for assessing depressive symptoms and screening for depression in Portuguese persons with cognitive impairment. This knowledge adds to a growing body of literature exploring the psychometrics of the GDS in various settings globally and, specifically, contributes to the growing knowledge of the Portuguese versions of the GDS-15 and GDS-27. 

## Figures and Tables

**Table 1:** Sociodemographic, clinical, and neuropsychological characteristics of study participants.

Sociodemographic, Clinical, and Neuropsychological Characteristics	Total Sample (*n* = 117)	Participants with Depression (*n* = 20)	Participants without Depression (*n* = 97)
Gender (number): male/female	27/90	3/17	24/73
Age (years): mean ± SD (range)	83.66 ± 7.47 (65–101)	83.75 ± 7.92 (69–101)	83.64 ± 7.42 (65–97)
Education level (%): - up to 2 years - between 3 to 6 years - 7 years or more	35.1% 58.1% 6.8%	55.0% 45.0% -----	30.9% 60.8% 8.2%
Marital status (%): - with partner - without partner	17.1% 82.9%	10.0% 90.0%	18.6% 81.4%
Type of institution attended (%): - residential structure/nursing home - adult day center - home support services - social center - other	76.1% 11.1% 2.6% 2.6% 7.7%	75.0% 20.0% ----- 5.0% -----	76.3% 9.3% 3.1% 2.1% 9.3%
Type of neurocognitive disorder diagnosis: - Vascular Neurocognitive Disorder - Neurocognitive Disorder due to Alzheimer Disease - Neurocognitive Disorder due to Parkinson Disease - Unspecified Neurocognitive Disorder - Neurocognitive Disorder due to traumatic brain injury - other (substance/medication induced or due to another medical condition)	24.8% 23.9% 6.8% 36.8% 4.3% 3.4%	15.0% 25.0% 10.0% 45.0% ----- 5.0%	26.8% 23.7% 6.2% 35.0% 5.2% 3.1%
Cognitive status: MMSE (score): mean ± SD (range)	18.89 ± 3.73 (9–27)	19.15 ± 2.96 (13–24)	18.84 ± 3.88 (9–27)

Abbreviations: BDI: Beck Depression Inventory; DSM-5: Diagnostic and Statistical Manual of Mental Disorders, 5th edition; GDS: Geriatric Depression Scale; MMSE: Mini-Mental State Examination.

**Table 2:** Results on the GDS questionnaires for the subsample of depressed and non-depressed participants.

DSM-5 Diagnostic Criteria	Mean ± SD (Range)	
	GDS-15	GDS-27
with depression (*n* = 20)	11.05 ± 2.01 (7–14)	20.45 ± 2.89 (16–25)
- mild depression (*n* = 10)	10.40 ± 1.43 (9–13)	19.70 ± 3.09 (16–25)
- moderate depression (*n* = 7)	11.00 ± 2.52 (7–13)	20.43 ± 2.64 (16–24)
- severe depression (*n* = 3)	13.33 ± 0.58 (13–14)	23.00 ± 1.73 (21–24)
without depression (*n* = 97)	6.99 ± 3.65 (0–15)	12.80 ± 6.50 (1–27)

Abbreviations: DSM-5: Diagnostic and Statistical Manual of Mental Disorders, 5th edition; GDS: Geriatric Depression Scale.

**Table 3:** Sensitivity, specificity, and Youden Index of Geriatric Depression Scale with 27 and 15.

GDS-27				GDS-15			
Cut-Off Point	Sensitivity	Specificity	Youden Index	Cut-Off Point	Sensitivity	Specificity	Youden Index
1.50	100%	3%	0.031	0.50	100%	1%	0.01
2.50	100%	4%	0.041	1.50	100%	5%	0.05
3.50	100%	5%	0.052	2.50	100%	14%	0.14
4.50	100%	13%	0.134	3.50	100%	23%	0.23
5.50	100%	20%	0.196	4.50	100%	29%	0.29
6.50	100%	22%	0.216	5.50	100%	39%	0.39
7.50	100%	24%	0.237	6.50	100%	43%	0.43
8.50	100%	29%	0.289	7.50	95%	52%	0.47
9.50	100%	35%	0.351	**8.50**	**90%**	**62%**	**0.52**
10.50	100%	40%	0.402	9.50	75%	73%	0.48
11.50	100%	42%	0.423	10.50	55%	81%	0.36
12.50	100%	45%	0.454	11.50	50%	90%	0.40
13.50	100%	51%	0.505	12.50	35%	93%	0.28
14.50	100%	59%	0.588	13.50	5%	97%	0.02
**15.50**	**100%**	**63%**	**0.629**	14.50	0%	99%	−0.01
16.50	90%	68%	0.580				
17.50	80%	75%	0.553				
18.50	70%	79%	0.494				
19.50	60%	85%	0.445				
20.50	50%	86%	0.356				
21.50	40%	88%	0.276				
22.50	25%	93%	0.178				
23.50	25%	96%	0.209				
24.50	5%	98%	0.029				
26.00	0%	99%	−0.010				

Items in bold: values of cut-off point, sensitivity, and specificity for the maximal Youden Index.

## Data Availability

The data that support the findings of this study are available from the corresponding author upon reasonable request. The data are not publicly available due to privacy/ethical restrictions.
